# Best Practices for Working with Latinx Older Adults in Mental Healthcare

**DOI:** 10.1093/geroni/igab046.3104

**Published:** 2021-12-17

**Authors:** Kendall Weber, Lisa Stone

**Affiliations:** 1 University of Colorado at Colorado Springs, University of Colorado at Colorado Springs, Colorado, United States; 2 University of Colorado Colorado Springs, Colorado Springs, Colorado, United States

## Abstract

**Background:**

The number of older adults in the United States is growing rapidly. The percentage of individuals from ethnic minority groups that make up this population is also rapidly increasing, with Latinx older adults comprising the fastest growing subgroup. However, Latinx older adults historically underutilize mental health services, in large part due to the lack of culturally sensitive and informed care provided by mental health professionals (de Guzman et al., 2015). However, to date, comprehensive, evidence-based best practices for mental healthcare for Latinx older adults do not exist. Method: A literature review was conducted of research on the developmental, social, cognitive, biological, and affective bases of behavior among Latinx older adults.

**Results:**

Taking an integrated, evidence-based psychological approach with cultural considerations, we found that the literature could broadly be organized into six best practice guidelines. We propose assessing for and incorporating the following topics into mental health treatment of Latinx older adults: immigration status, acculturation, attitudes towards mental health, physical and cognitive health disparities, discrimination, and unique preferences for care structure in later life.

**Discussion:**

These guidelines are intended to represent basic principles to incorporate into practice and do not represent an exhaustive list of factors to consider for a heterogenous group of older adults. Instead, the six, empirically-based guidelines proposed in this study can serve as a starting point for increasing mental health providers’ awareness of the unique experiences of Latinx older adults, with the aim of improving the experience of this historically underserved population in mental healthcare treatment.

